# From stable teamwork to dynamic teaming in the ambulatory care diagnostic process

**DOI:** 10.1515/dx-2024-0108

**Published:** 2024-10-21

**Authors:** Scott I. Tannenbaum, Eric J. Thomas, Sigall K. Bell, Eduardo Salas

**Affiliations:** The Group for Organizational Effectiveness, Inc., Albany, NY, USA; The UTHealth-Memorial Hermann Center for Healthcare Quality and Safety, McGovern Medical School at UTHealth, Houston, TX, USA; Department of Medicine, Beth Israel Deaconess Medical Center, Boston, MA, USA; Department of Psychological Sciences, Rice University, Houston, TX, USA

**Keywords:** ambulatory care, diagnostic process, teaming, teamwork

## Abstract

Dynamic teaming is required whenever people must coordinate with one another in a fluid context, particularly when the fundamental structures of a team, such as membership, priorities, tasks, modes of communication, and location are in near-constant flux. This is certainly the case in the contemporary ambulatory care diagnostic process, where circumstances and conditions require a shifting cast of individuals to coordinate dynamically to ensure patient safety. This article offers an updated perspective on dynamic teaming commonly required during the ambulatory diagnostic process. Drawing upon team science, it clarifies the characteristics of dynamic diagnostic teams, identifies common risk points in the teaming process and the practical implications of these risks, considers the role of providers and patients in averting adverse outcomes, and provides a case example of the challenges of dynamic teaming during the diagnostic process. Based on this, future research needs are offered as well as clinical practice recommendations related to team characteristics and breakdowns, team member knowledge/cognitions, teaming dynamics, and the patient as a team member.

## Introduction

As healthcare has become more complex and fragmented, the demand for effective teamwork has intensified. From the moment a patient presents with new symptoms, their care relies heavily on coordinated teamwork. While teamwork competencies are required for safe diagnosis [1], research has often focused on teams with set roles and stable membership, with team members working on tasks synchronously and in close proximity to one another, or what can be thought of as “stable teamwork.” However, this no longer describes how collaboration occurs in many healthcare settings, especially in the ambulatory care diagnostic process.

Unfortunately, the profession has experienced slow, inadequate progress in diagnostic safety over the past decade [2]. Ambulatory care diagnostic errors and delays occur in 5 % of visits and are estimated to impact 12 million patients in the U.S. annually [3], 4]. In one U.S. study, half of all patients who were undiagnosed after an initial primary care visit remained undiagnosed 12 months later [5]. The gravity of this global problem necessitates an updated perspective that reflects the type of dynamic teaming commonly required during the ambulatory care diagnostic process.

During the ambulatory care diagnostic process (DxP), safety hinges on communication and coordination among a diverse group of individuals, including patients, family members, primary care providers, specialty physicians, nurses, radiologists, pathologists, care partners, medical interpreters, case managers, and others. Team members must collaborate at different stages of the diagnostic process with people they may have never met. They need to share health information but don’t necessarily hold a shared understanding of the patient’s diagnostic journey. As a case progresses, new people may join the “diagnostic team,” while others may complete a task or two and step aside. Patient and family contributions are increasingly recognized as a vital part of the diagnostic process, and while they can provide first-hand knowledge of what the patient has experienced, they typically have no medical expertise and may not be consistently recognized as team members per se.

Risks in this complex, fragmented, and dynamic context differ from those faced by teams with more stable roles and membership. To enhance safety in the DxP, we must consider how dynamic teaming differs from stable teamwork. Based on Edmondson’s concept of teaming as “teamwork on the fly” [6], teaming is particularly relevant when the fundamental structures of a team, such as membership, priorities, tasks, modes of communication, and team member location, are in flux. While teaming implicitly suggests shifting conditions, we refer to it here as “dynamic teaming” to emphasize how it differs from stable teamwork. Dynamic teaming is required when people must coordinate with one another in a fluid context, as is often the case during the ambulatory diagnostic process.

This article draws upon team science to clarify the characteristics of dynamic diagnostic teams and common risk points in the teaming process, surface the practical implications of these risks, suggest potential opportunities for improving teaming, and identify research needs.

## Characteristics of ambulatory DxP teams

### How teams differ

**Figure 1:**
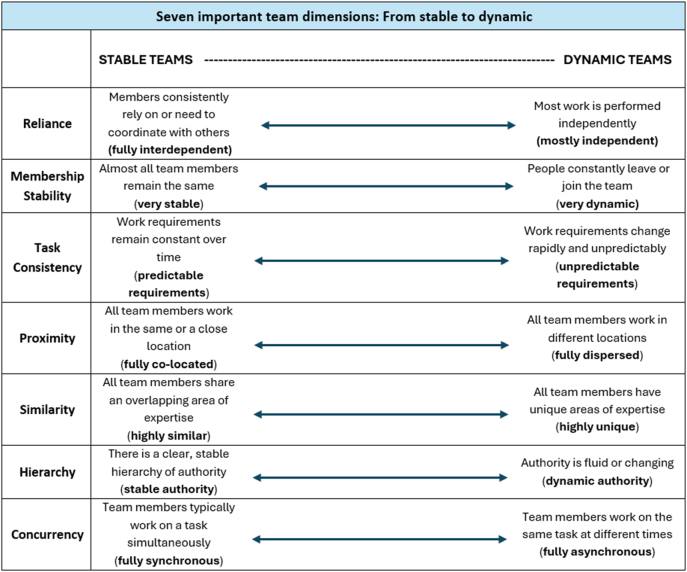
Seven important team dimensions: from stable to dynamic based on Tannenbaum and Salas [7].

Teams are not all the same. A comprehensive review of the team effectiveness research literature [7] identified five dimensions or continua along which teams commonly vary: 1) Reliance, 2) Membership stability, 3) Task consistency, 4) Proximity, and 5) Similarity. In addition, two other dimensions can help differentiate stable and dynamic teams: 6) Hierarchy and 7) Concurrency (Figure 1). As shown in Figure 1, the left side of each continuum describes a more stable context, while the right side reflects a more dynamic one. Because different teaming risks arise at various points along each continuum, a team’s profile has implications for how it should prepare and perform together.

The following case describes an ambulatory diagnostic team involved in a new cancer diagnosis (case adapted from a previously published case [8]). As the case progresses, consider: 1) Who is part of this diagnostic team? 2) Are these individuals aware of their own and others’ roles? 3) Where does this team fall along the seven dimensions? 4) What did this team do well? 5) What would help this team operate more effectively?

Maria Gomez is a 49-year-old Spanish-preferring woman with hypertension, diabetes, and hypercholesterolemia. During a routine visit involving an interpreter, Ms. Gomez reported a breast lump to her primary care provider (PCP), Dr. Bloom. Dr. Bloom noted a cystic area that was not different from the rest of the breast tissue. Since the patient was near the start of her menstrual cycle, Dr. Bloom recommended she be reexamined at a later date. Dr. Bloom subsequently went on leave, and Dr. May assumed the patient’s care. Ms. Gomez saw Dr. May several times for high blood pressure. Some visits included an interpreter, and some did not. Ms. Gomez did not mention the breast lump, assuming the doctor would resume Dr. Bloom’s plan if he were concerned, and Dr. May did not notice this problem in her chart. Six months later, Ms. Gomez saw Dr. Ruiz, her Spanish-speaking obstetrician/gynecologist (OB-GYBN), and mentioned the breast lump, noting it had gotten bigger. Dr. Ruiz ordered a mammogram, which confirmed the mass. Based on radiology protocol, the mammogram was followed by an ultrasound and biopsy performed by the radiologist, Dr. Smith. The pathologist confirmed breast cancer, and this critical result was called to Dr. Smith’s office. The final pathology report was sent to Drs. Smith, Ruiz, and May. Unaware of the breast lump, Dr. May was surprised by the recent events and findings. Ms. Gomez was referred to oncology to discuss a treatment plan. Upset about the results, she wondered if the breast cancer could have been diagnosed earlier.

In this case, the diagnostic team consisted of at least six individuals, including the patient, performing a mix of independent tasks (i.e., could be completed alone) and interdependent tasks (i.e., people rely on one another.) Team membership was fairly dynamic over time. They dealt with evolving requirements, were mainly dispersed, often worked asynchronously, and possessed unique areas of expertise. Perceived hierarchy and a lack of consistent interpreters may have inhibited the patient’s openness when communicating with Dr. May, compared to her language-concordant OB-GYN physician. While some team members had clear roles and procedures (such as the radiology protocol), others (like the primary care team) lacked standardized pass-offs and expectations, leading to preventable delays. Overall, this team needed to engage in dynamic teaming more than stable teamwork.

While diagnostic teams are not uniform, we suggest the Gomez team profile is not atypical in complex diagnostic processes. Data suggest the “Big Three” diseases – cancer, cardiovascular disease, and infectious disease – account for about three-fourths of serious misdiagnosis-related harms in the U.S [9], 10]. and often involve numerous individuals interacting in similar dynamic teaming conditions. Contrast this with the profile of a team that assembles to perform surgery or rounds on patients in an intensive care unit. Those teams have more stable clinical roles and relatively consistent task requirements and work in close physical proximity to one another.

In the ambulatory care DxP, teaming occurs on both an as-needed and a continuous basis, with coordination demands often triggered by an anticipated or emergent task requirement. Team members must balance multiple objectives, shift from one situation to another, integrate perspectives of multiple people and disciplines, communicate across dispersed locations yet work asynchronously on different aspects of the diagnostic process, quickly process complex information, and do all this with limited pre-planned coordination [6]. An inferred hierarchy may exist, but authority can be diffused or fluid as a case proceeds.

## Teaming risks and consequences

**Figure 2:**
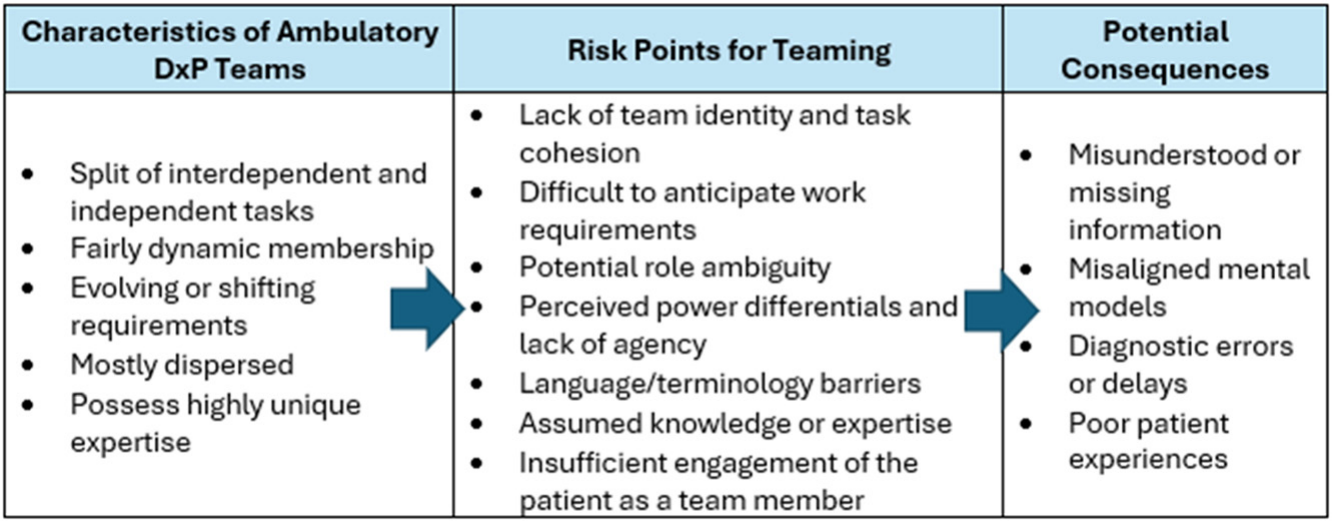
Characteristics, teaming risk points, and possible adverse outcomes in ambulatory diagnostic teams.

Given the dynamic nature of DxP teams, there are several emergent but predictable teaming risk points (as seen in the Gomez case), which, if not attended to, can result in adverse outcomes, as indicated in Figure 2.

### Lack of team identity and cohesion

People with a common team identity tend to share more information with one another [11]. Because a diagnostic team has dynamic membership and is composed of people with differing expertise, team members may be more likely to identify with their profession or functional area than with the transient team formed to diagnose a patient. Individuals in multi-disciplinary teams tend to share a stronger sense of cohesion (a shared attraction, bonding, or sense of pride [12]) with people from their discipline, and different professions or sites may prioritize work differently [13]. Some people involved in the diagnostic process may not consider themselves part of the diagnostic team and, as a result, may focus narrowly on their own tasks rather than viewing their role within the context of the broader diagnostic teaming process. When team identity and cohesion are low, intentional integration of team members through cross-discipline “boundary spanning” (actions that help bridge and connect potentially disparate groups) is needed to ensure ample coordination and cooperation as a case proceeds [14]. In the Gomez case, one may contrast the teaming in the hospital-based primary care practice with that in the OB-GYN clinic. Ms. Gomez acted as a “boundary spanner” when she bridged the gap between her PCP’s office and her OBGYN physician by bringing the breast lump to the latter’s attention, although she may not have known or been told she was part of the diagnostic team. The auto-notification of critical test results to the ordering clinician, the OB-GYN physician, and the PCP provided a systems-based boundary-spanning mechanism for information sharing.

### Difficult to anticipate work requirements

The diagnostic process does not always progress in a predictable, linear fashion – diagnostic work is naturally emergent. New information about the patient creates evolving tasks, and expertise, timing, and collaboration demands, which can differ from case to case. A rote process or fully repeatable routine or timeline is rarely feasible. Team members engaged in dynamic teaming are less able to anticipate and prepare for coordination needs than a stable team. The tasks and risks are inherently less predictable, such as the missed step of informing Dr. May (and the patient) of the need to follow up on the patient’s breast lump or the unshared expectation that Dr. May would review older notes and information that did not pertain to the patient’s more immediate needs (blood pressure management). As a result, coordination can suffer, avoidable delays may occur, and the patient experience may feel disjointed rather than unified.

### Potential role ambiguity

As the diagnostic process progresses, if requirements change or new people get involved, it is easy for role ambiguity (uncertainty) or role conflict (disagreement) to arise. Patients and their care partners may have difficulty knowing their own roles and responsibilities (e.g., what to do if a lump does not go away), especially if they are not treated as diagnostic team members or informed of their potential roles, as in the Gomez case. In addition, they may not know how different providers relate to one another or who is “in charge” [15]. Clinicians may not always be clear about who “owns” a particular task. Team members may have different assumptions about who is responsible for ordering a test or updating the patient about test results, so the patient might receive two different updates, which can generate confusion, or no update at all. Role ambiguity can adversely affect team performance [16], resulting in delays, missed steps, and a degraded patient experience. In a dynamic setting that spans time and geographic space (through different healthcare encounters), it is more challenging to maintain a mutual understanding among team members about patient status, provider and patient responsibilities, and updated priorities. Yet this shared understanding among team members – i.e., a “shared mental model” has been linked to team performance [17], 18].

### Perceived hierarchy and lack of agency

Providers may enter the diagnostic teaming process with some implicit assumptions about authority and their power relative to others. For example, if a resident believes the attending will dismiss their perspective, psychological safety (a shared belief that it is acceptable to ask questions, offer ideas, and provide input) declines, and they are less likely to speak up [19], 20]. Important information may not be conveyed, and an opportunity to provide backup and avert an error may be lost. Similarly, if a patient like Ms. Gomez perceives little control or agency over how her care will proceed, she may be reluctant to ask a question, raise a concern, or share something they noticed. Patients are arguably perceived as lowest in the medical hierarchy, which may inhibit their willingness to speak up or ask questions of the clinical team [21]. They can default to being passive healthcare recipients rather than active team members, especially when cultural, social, or language barriers discourage speaking up, as in this case. Research has shown that engaging multiple people in the diagnostic and patient care process can enhance decision-making quality [22], 23] through the “wisdom of crowds.” However, when a perceived hierarchy inhibits active participation, it heightens the risk of error and delays.

### Language and terminology barriers

Diagnostic teams often include individuals from different professions, and healthcare providers are prone to using jargon that is specific to their field, creating professional language barriers. Some patients may (literally) not speak the same language as their healthcare providers, and very few speak or fully understand medical jargon. When professional or literal language barriers are coupled with a reluctance to ask questions or admit when something is not clear, a team is increasingly prone to communication breakdowns. This can be exacerbated by an inconsistent use of interpreters, which not only threatens accurate information transfer but also sends an implicit message to the patient that they are not expected to be active contributors.

### Assumed knowledge or expertise

When expertise and knowledge are distributed across team members, the team needs to maintain an awareness of “who knows what,” referred to as a transactive memory system [24], 25]. Unfortunately, when team membership is dynamic and people work in different locations, they are less likely to know exactly who knows what. For example, if remote prostate cancer was treated years ago in another organization and not mentioned in more recent notes, or if an organization implements a new EHR that does not capture accurate dates of past events during data migration, clinicians may lack the information they need. In the Gomez case, assumed (but lacking) knowledge of the breast lump delayed the diagnosis. Lack of interoperability between healthcare organizations and faulty data transfer or sharing can put transactive memory at risk, with potentially negative consequences.

The assumptions people make about distributed knowledge can contribute to this risk. Team members often erroneously believe that another team member holds important information, that they would share if necessary. This type of false assumption has been referred to as illusory transactive memory [26]. Providers may also incorrectly assume that others possess the same knowledge they do about the patient, recent test results, or what the results mean, so they don’t feel compelled to communicate that information (an assumption we refer to as “everybody knows”). For example, if a neurologist is evaluating a patient with a remote history of prostate cancer for a new brain lesion, the PCP and patient may assume that the neurologist is aware of the cancer history and would thus include metastatic prostate cancer as a possible diagnosis.

Information that is known throughout a team (“shared information”) is more likely to be referenced and acted upon than information known by only one or two team members (“unique information”) [27]. This is another reason why explicitly sharing unique information is critical for patient safety.

### Insufficient engagement of the patient

A team already grappling with how to coordinate dynamic membership and evolving requirements may consciously or subconsciously refrain from engaging patients due to time demands. This is a missed opportunity since patients can help facilitate information sharing and boundary spanning, especially when multiple providers or centers are involved in their care [28]. A lack of patient engagement can increase the risk of missed and delayed diagnoses by making patients less alert for potential breakdowns and less likely to volunteer their unique knowledge. To optimize performance, a diagnostic team, including the patient, must maintain a shared mental model of roles and responsibilities, emergent facts, contingency plans, how they will communicate, and the timing of the next steps. This was a key missing step in the Gomez case, which may be attributed to time demands, lack of an interpreter, faulty information transfer, or other factors. Everyone involved needed to be alert for potential risk points before they resulted in a problem. Unfortunately, shared mental models are less likely to develop when the patient is not seen as a team member.

## Recommendations for clinical practice

Viewing the ambulatory diagnostic process through a dynamic teaming lens can lead to insights for research and practice. Although research questions remain, the extant literature provides a basis for a few preliminary clinical practice suggestions. Below are nine recommendations clustered into four categories.

### Team characteristics and teaming breakdowns

1. Create an assessment tool and “playbook” with strategies specifically tailored for diagnostic teams, consistent with their degree of reliance, proximity, hierarchy, etc.
2. Build teaming-related warning prompts or support strategies into electronic communication tools for patients and clinicians to help diagnostic teams avert or mitigate common breakdowns.

### Team member knowledge/cognitions

(3) Train clinicians to build inter-positional knowledge awareness (a general understanding of others’ roles on a team), starting in early interprofessional healthcare education, while also beginning to build student appreciation of the patient as a team member [29].
(4) Develop checklists and discussion guides to structure, clarify, and sustain a team’s shared mental model. These would be different from diagnostic checklists, which have demonstrated mixed efficacy [30]. Instead, they focus specifically on teaming needs and risks during the diagnostic process.

### Teaming dynamics

(5) Establish guidelines for using electronic communications during the diagnostic teaming process, including the use of the patient portal to build engagement.
(6) Provide training or coaching to diagnostic team leaders to help them promote psychological safety and mitigate the negative aspects of perceived hierarchy.

### Patient as team member

(7) Offer multilingual guidance and tipsheets for patients and family members as contributing team members during the diagnostic process. Identify and address barriers to engagement, including the patient portal. Normalize and meaningfully respond to patient contributions to the diagnostic process.
(8) Demonstrate consistent and visible leadership support for patient engagement, and incorporate patient insights into educational settings such as ground rounds, morbidity and mortality conferences, etc. Involve Patient and Family Advisor Councils in patient education resource development, governance committees, and related research efforts.
(9) Train providers to uncover patient preferences and prepare them to work with patients who want to be part of the team and those who do not.

## Limitations and future research

While there is an increasingly large body of research on teamwork, far more is known about teams working in relatively stable than in dynamic environments. Research is needed to clarify how dynamic teaming manifests during the diagnostic process. This contention is supported by a recent systematic review of diagnostic safety research involving 97 researchers and 42 stakeholders. Six of the top 15 diagnostic safety research priorities identified pertained directly to teamwork [31].

Future research should address common limitations in the research literature. Given the dynamic nature of diagnostic teaming, future studies would benefit from greater use of longitudinal designs that capture what happens as the diagnostic process unfolds over time, with data gathered from both providers and patients. Qualitative or mixed methods should be employed to better understand teaming nuances and dynamics. Longitudinal, multi-source, multi-methods research could help address a wide range of questions, including the five high-priority research questions we identify below:

1. Which teaming barriers and breakdowns emerge most frequently during the diagnostic process?
2. What are the similarities and differences in how healthcare providers and patients view teaming in the diagnostic process? How do patients at risk of healthcare disparities perceive the diagnostic process, and how can organizations equitably support their role as diagnostic team members?
3. How can we best enable patients and their families to contribute to and maintain a shared mental model with clinical members of the diagnostic team? How do electronic record systems affect the formation of shared mental models?
4. Which teaming tools and interventions can enhance coordination during the diagnostic process? What evidence-based teaming practices should be tested with diagnostic teams?
5. Which teaming behaviors during the diagnostic process most significantly impact clinical outcomes such as errors?

## Conclusions

Dynamic teaming is increasingly how healthcare collaboration occurs. This is certainly the case in the contemporary ambulatory care diagnostic process, where circumstances and conditions require a shifting cast of individuals to coordinate dynamically to ensure optimal patient care and safety. This article addresses the need for an updated perspective on dynamic teaming during the ambulatory diagnostic process. It highlights how the characteristics of DxP teams can create common teaming risks and considers the role of providers and patients in averting adverse outcomes. Research is needed to explicate further the nuances and drivers of dynamic teaming so that inherent risks and opportunities may be anticipated and addressed.
